# Supplementary material for: Histone H1 Limits DNA Methylation in Neurospora crassa
Source: G3 (Bethesda). 2016 May 6;6(7):1879–89. doi: 10.1534/g3.116.028324 (PMC4938642; doi:10.1534/g3.116.028324)
Supplement: Supplemental Material [file supp_6_7_1879__index.html]

Histone H1 Limits DNA Methylation in Neurospora crassa — Supplemental Material 

# Histone H1 Limits DNA Methylation in *Neurospora crassa*

## Supplemental Material for Seymour *et al.*, 2016

**Files in this Data Supplement:**

- Figure S1 - Metaplots depict the average sequencing depth on the + strand across all *N. crassa* genes or for gene expression groups. (.pdf, 327 KB)
- File S1 - BROAD *Neurospora crassa* or74a 12 transcripts. (.zip, 661 KB)
