# Supplementary material for: Histone H1 Limits DNA Methylation in Neurospora crassa
Source: G3 (Bethesda). 2016 May 6;6(7):1879–89. doi: 10.1534/g3.116.028324 (PMC4938642; doi:10.1534/g3.116.028324)
Supplement: Supplemental Material [file supp_g3.116.028324_FigureS1.pdf]

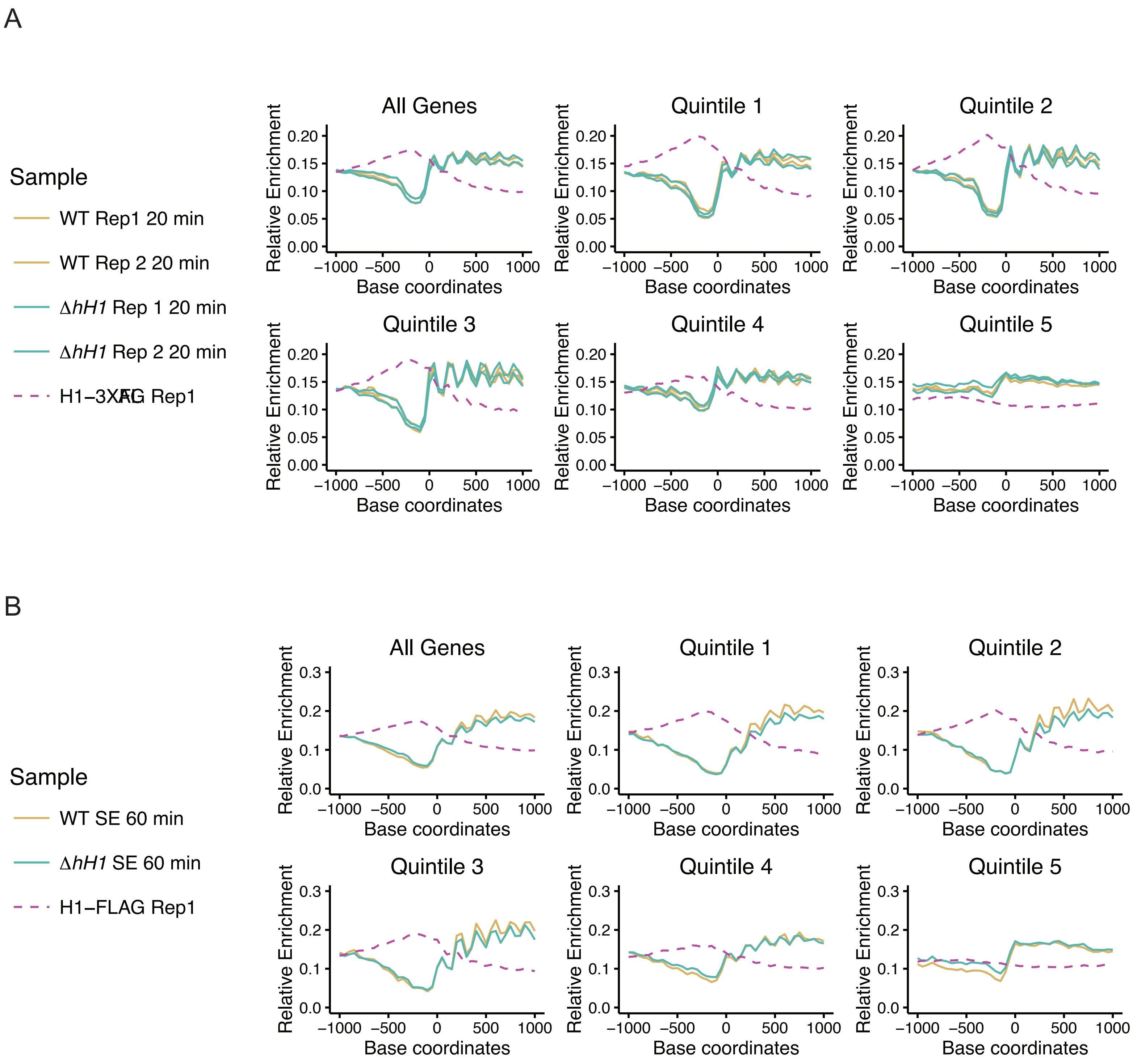

Figure S1 Metaplots depict the average sequencing depth on the + strand across all *N. crassa* genes or for gene expression groups. For expresson group analysis, all *N. crassa* genes were ranked by expression level and split into quintile groups, ranging from highest expression (Quintile 1) to lowest expression (Quintile 5).(A) Data from paired-end MNase-seq from a 20 minute MNase digest. Plots show data from two biological replicates each for wildtype and Δ*hH1*. (B) Data from single-end MNase-seq from a 60 minute MNase digest. For all plots, the coverage of + strand reads from an H1-3XFLAG ChIP-seq experiment is shown as a dashed line.
